# Comparing Real-time Versus Delayed Video Assessments for Evaluating ACGME Sub-competency Milestones in Simulated Patient Care Environments

**DOI:** 10.7759/cureus.2267

**Published:** 2018-03-04

**Authors:** Robert Isaak, Marjorie Stiegler, Gene Hobbs, Susan M Martinelli, David Zvara, Harendra Arora, Fei Chen

**Affiliations:** 1 Department of Anesthesiology, University of North Carolina School of Medicine; 2 Department of Neurosurgery, University of North Carolina School of Medicine

**Keywords:** video assessment, milestone assessment, osce, anesthesiology, simulation, graduate medical education

## Abstract

Background

Simulation is an effective method for creating objective summative assessments of resident trainees. Real-time assessment (RTA) in simulated patient care environments is logistically challenging, especially when evaluating a large group of residents in multiple simulation scenarios. To date, there is very little data comparing RTA with delayed (hours, days, or weeks later) video-based assessment (DA) for simulation-based assessments of Accreditation Council for Graduate Medical Education (ACGME) sub-competency milestones. We hypothesized that sub-competency milestone evaluation scores obtained from DA, via audio-video recordings, are equivalent to the scores obtained from RTA.

Methods

Forty-one anesthesiology residents were evaluated in three separate simulated scenarios, representing different ACGME sub-competency milestones. All scenarios had one faculty member perform RTA and two additional faculty members perform DA. Subsequently, the scores generated by RTA were compared with the average scores generated by DA. Variance component analysis was conducted to assess the amount of variation in scores attributable to residents and raters.

Results

Paired t-tests showed no significant difference in scores between RTA and averaged DA for all cases. Cases 1, 2, and 3 showed an intraclass correlation coefficient (ICC) of 0.67, 0.85, and 0.50 for agreement between RTA scores and averaged DA scores, respectively. Analysis of variance of the scores assigned by the three raters showed a small proportion of variance attributable to raters (4% to 15%).

Conclusions

The results demonstrate that video-based delayed assessment is as reliable as real-time assessment, as both assessment methods yielded comparable scores. Based on a department’s needs or logistical constraints, our findings support the use of either real-time or delayed video evaluation for assessing milestones in a simulated patient care environment.

## Introduction

The Next Accreditation System (NAS) of the Accreditation Council for Graduate Medical Education (ACGME) requires training programs to evaluate residents on “milestones” that relate to competency-based outcomes (e.g., knowledge, skills, and attitudes) throughout residency [1]. One of the many challenges of milestone assessment is the unpredictable frequency in which particular patient care events occur. The timing and frequency of clinical encounters during which residents will have an autonomous opportunity to demonstrate competency of milestones is very difficult to predict and observe. For instance, “Patient Care 5 – Crisis Management” of the Anesthesiology Milestones Project states that a resident at level 4 will “assume increasing responsibility for leadership of a crisis response team” [2]. This milestone is particularly challenging to assess since critical events are rare and often unpredictable with regards to their timing of occurrence. As a result, it is difficult to observe a resident in the midst of a crisis in a predictable and timely fashion. Additionally, patient care needs during a crisis may supersede the attending faculty member’s ability to provide critical observation and evaluation of the resident in the leadership of the team. In order to overcome these barriers and to reliably and consistently create comparable opportunities to evaluate these kinds of milestones for all of our residents, some residency programs utilize simulation and standardized case scenarios [3-4].

Simulation is a reliable method [5-9] for assessing the performance of clinical skills in trainees. Additionally, simulation-based assessments have the benefit of removing the ethical commitment to provide the most timely and appropriate patient care by the most experienced clinician (e.g., the supervising attending physician). Subsequently, simulation-based assessments allow trainees to demonstrate the presence, or lack, of clinical competency without the potential of causing patient harm. The use of simulation for assessment can come in two forms; formative (“low-stakes”) or summative (“high-stakes”) assessments. Formative assessments occur during the learning process of a resident (e.g., throughout the years of residency training) with the goal of modifying the teaching and learning activities that can improve the learner’s skill set and knowledge base. Conversely, summative assessment serves to measure the outcome of the learning activities at the conclusion of a resident’s training program. The use of objective structured clinical exams (OSCEs) during residency training can serve the role of formative assessment, summative assessment, or both, depending on the purpose of obtaining the assessments.

Medical training programs have been using simulation for more than two decades in the context of OSCE to address the issue of reliability, repeatability, and objectivity in the assessment of their trainees [10-11]. An OSCE allows for the incorporation of high-fidelity simulation equipment, standardized patient actors, and objective scoring procedures to reliably and consistently replicate scenarios that allow residents to demonstrate a variety of skills [12-14]. Since the measurement of ACGME milestones is simultaneously intended to give feedback regarding the effectiveness of learning activities and to measure outcomes of achievement through the continuum of residency training, the use of an OSCE to provide formative, as well as summative assessment, is very valuable. However, summative assessment of milestones as a “high stakes” assessment is a bit overstated since milestones are merely intended to serve as benchmarks and aspirational goals for residents upon completion of their training, not as a tool for judging whether a resident will be permitted to graduate from their training program.

When evaluating residents’ clinical competency in an OSCE or simulated patient care scenario, assessors typically perform real-time assessments (RTA) or contemporaneous live observation [15-17]. RTA is also the default method of resident assessment for traditional clinical evaluations (e.g., daily evaluations, rotation evaluations, clinical milestone evaluations). Similarly, RTA is the most common method of evaluating residents’ clinical skills during traditional supervised patient care activities in real clinical environments. However, when a large number of residents require milestone evaluations on several OSCE-based scenarios, RTA can be very resource intensive and logistically difficult to schedule. This is especially true with regards to the time commitment needed from attending physician faculty members. For most OSCE scenarios at the graduate medical education level, content expertise from the evaluator is necessary; hence, a clinical faculty member – rather than an education specialist, standardized patient, or standardized clinician – is required to complete the evaluation. Additionally, due to the need for efficiency during OSCE sessions, faculty members need to complete OSCE performance evaluations in a tightly scheduled time frame when performing RTA, which can be cognitively demanding to the evaluator. As a practical matter, in many residency training programs, the number of residents requiring evaluation and the number of simulation-based milestone scenarios needed for assessment will likely overwhelm the supply of faculty available for RTA, especially in large-scale simulation-based assessments. One potential solution to these issues is to video-record the simulated scenarios and allow faculty to perform delayed assessments (DA). 

We hypothesized that DA scores, based on video review, reliably represent RTA scores in simulated patient care environments. In order to test this hypothesis, our study compared the scoring between RTA and DA of residents’ ACGME sub-competency milestone performance in simulated encounters using different raters for each approach.  

## Materials and methods

Sample

Each post-graduate year (PGY) 2-4 resident (n = 41) completed 3 separate simulated clinical scenarios (Table 1) focused on a different anesthesiology sub-competency milestone. A few of the resident’s scenarios had technical issues related to the video recording (e.g. camera not started on time) during their scenarios (two for Case 2 and two for Case 3) and therefore could only be scored via RTA. Those individual resident scenarios were excluded from the study, yielding data from 41 residents for analysis of Case 1 and 39 residents for analysis of Cases 2 and 3.

**Table 1:** Clinical Scenarios and Milestone Assessed

Case Number	Scenario Title	Milestone Assessed	Simulation Technique(s)
1	Trauma Resuscitation	Patient Care 5 (PC5): Crisis management	High-fidelity computerized mannequin and standardized patient actor
2	“Can’t Intubate/Can’t Ventilate”	Patient Care 8 (PC8): Airway management	High-fidelity computerized mannequin, standardized patient actor, and partial task trainer
3	Consent for a Jehovah’s Witness patient	Professionalism 1 (PROF1): Responsibility to patients, families, and society	Standardized patient actor

Measures

OSCE Scenarios

The OSCE session assessed 41 anesthesiology residents (PGY2-PGY4), 13-15 residents per class, at the University of North Carolina over two separate days of administration in January 2015. The three simulation-based OSCE scenarios assessed sub-competency milestones addressing patient care and professionalism domains from the Anesthesiology Milestones Project [2]. Each scenario was 7-15 minutes in length. The department’s clinical competency committee and simulation committee selected sub-competency milestones that were deemed difficult to assess through routine direct clinical observation and were feasible to simulate in a standardized fashion. A thorough description of the process for designing the scenarios and a sample of the assessment worksheet has previously been described [18]. Briefly, the scenarios utilized multiple simulation modalities including low-fidelity standardized clinicians and patients, high-fidelity mannequins (Laerdal SimMan3G, Wappingers Falls, NY), procedural task trainers (Simulab, Seattle, Washington), or a mixture of modalities to most adequately assess the identified sub-competency milestone (Table 1). Although residents were familiar with the simulation environment as part of their usual educational activities, they were blinded to the milestones and scenarios being examined as well as to the content of the evaluation checklists prior to the OSCE session.

Raters Selection and Training

Nine raters participated in the study: three completing RTA and six completing DA. All of the raters were considered “core faculty members” of the residency program as they were all actively involved in the resident educational curriculum, specifically as simulation-based education instructors. All of the raters received training in two steps. First, a standardized email was sent to all of the raters that gave an overview of ACGME milestones, an explanation for using simulation to assess milestones, and details on the modified Delphi procedure taken to develop the behaviorally anchored analytical score sheets (Appendix 1) [19]. Second, each of the raters had an individual in-person meeting with the study primary investigator to review the score sheets and answer questions regarding the analytical items or the overall scoring process. All of the DA raters scored the scenarios individually during the three weeks immediately following the simulation sessions. DA raters were able to choose the time and place most convenient for them to complete the assessments (e.g., in their office during non-clinical hours). The primary reason for having one RTA rater was to simulate real-world conditions, in that having multiple content experts (i.e., clinical attending anesthesiologists) was not practical for our program due to our high clinical burden. 

Evaluation Checklists

Using a modified Delphi method [20], non-weighted evaluation checklists were developed, based on the milestones’ rubrics, for each scenario to standardize the assessment level of residents’ performance. The expert consensus of residents’ behavior on the checklists directly related to each portion of the milestone rubric and therefore represented objective markers of attainment for specific milestone levels. Raters were trained to score performance based on the behavioral checklist and not on their subjective assessment of performance. The scale of the OSCE performance measurements was 0 - 5 in half-point increments, corresponding to milestone levels 0 - 5. Appendix 1/Table 2 demonstrates an example of the conversion of an ACGME sub-competency milestone rubric into an analytical checklist for assessment in the simulated patient encounters.  

Procedures

All three OSCE scenarios were administered at the University of North Carolina Clinical Skills and Patient Simulation Center with fully re-created clinical care areas that simulated operating rooms and other perioperative locations. The center is equipped with high-fidelity patient mannequins and a multi-angle audio-visual recording system (CAE Healthcare Learning Space, Sarasota, FL) in each encounter area. Each of the residents’ simulated scenario performances was scored by three faculty raters, one rater via RTA and two raters via DA, using a secure streaming video source (CAE Healthcare Learning Space, Sarasota, FL). Each of the three scenarios had a different set of three faculty members performing the assessments, yielding a total of nine faculty members who served as assessors. All raters received training on evaluating milestone performance based on the provided rubrics and checklists.

Analysis

To determine if the DA scoring method produced scores equivalent to RTA, we used paired t-tests to compare the scores generated by the faculty assessing in real-time versus the average scores of the faculty members assessing via delayed recordings for each individual scenario. We further calculated intraclass correlation coefficients (ICC) of residents’ scores on the three scenarios to examine the agreement between DA and RTA in assessing the milestone competency. ICC was chosen over other reliability analysis methods because it permits the estimation of both the actual inter-rater reliability of the number of raters used in the study and the reliability of a single rater [21]. Additionally, if there were any missing values (e.g., audiovisual malfunction that led to a missing recording of a resident’s performance), then the score was managed by a two-way random ICC. To offer a more detailed perspective into the variance structure, the amount of variation in score associated with resident’s true milestone level and the rater as random-effects variables was also assessed. SAS 9.4 (SAS Institute Inc., Cary, NC) was used for data analysis.

## Results

**Figure 1:**
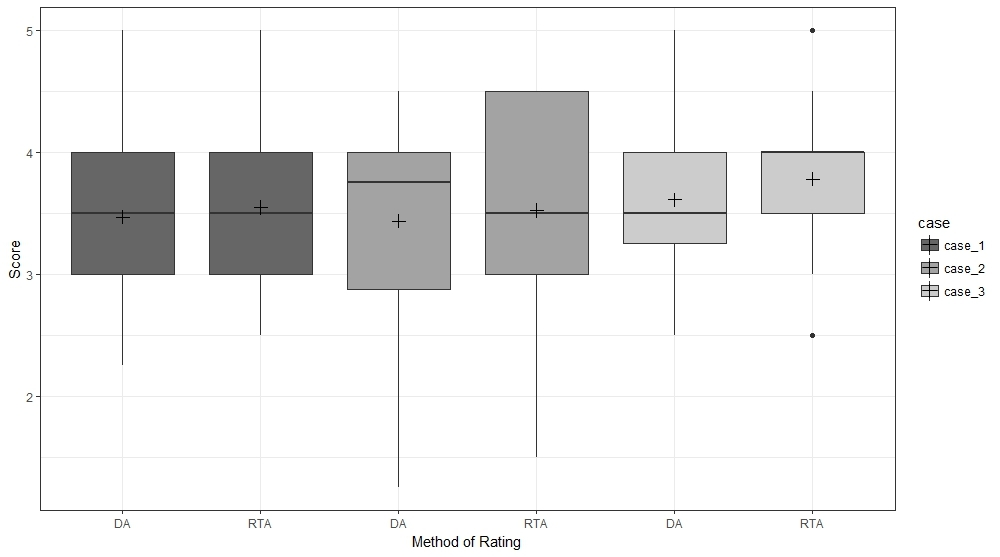
Score distributions of the cases Boxplots showing mean (+), median (central horizontal line), 25^th^ (lower end of the box), and 75^th^ percentile (upper end of the box) for scores given by assessment approach (delayed assessment (DA) versus real-time assessment (RTA)). The upper whisker represents scores larger than 75^th^ percentile but less than 1.5 times of the upper quartile. The lower whisker represents scores less than 25^th^ percentile but greater than 1.5 times of the lower quartile. The dots represent those outliers that are greater (or less) than 1.5 times of upper (or lower) quartile. Note: The DA score is the average of the score assessments from the two DA raters.

There were no major differences in the mean, median, 25th percentile, 75th percentile, or range of score distributions between the RTA raters and the DA raters who assessed the same scenario (Figure 1). Paired t-test results confirmed that there was no significant difference in scores between RTA and DA for any scenario (Table 3).

**Table 3:** Pair T-test Between Scores Based on Real Time and Averaged Delayed Assessments by Case Mean Diff: mean difference between delayed assessment (DA) and real-time assessment (RTA); SD: standard deviation; 95% CI LL: 95% confidence interval lower limit; 95%CI UL: 95% confidence interval upper limit

Case	n	Mean Diff (SD)	95%CI LL	95%CI UL	t	p
1	41	-0.08 (0.54)	-0.25	0.09	-0.95	0.35
2	39	-0.13 (0.47)	-0.28	0.02	-1.71	0.10
3	39	-0.19 (0.59)	-0.38	0.01	-1.96	0.06

The intraclass correlation coefficients for agreement between RTA scores and average DA scores were 0.67, 0.85, and 0.50 for scenarios 1, 2, and 3, respectively. As a result, the ICCs demonstrated good, excellent, and fair reliability, respectively [20]. Overall, the average of the delayed scores by two reviewers was shown to be similar to the RTA score (Table 4). Analysis of variance of the scores assigned by the three raters showed the largest contribution of variance came from residents’ true milestone competency level (44% to 75%), while the proportion of variance attributable to the rater was much smaller, ranging from 4% to 15% (Table 5).

**Table 4:** Intraclass Correlation Coefficients (ICC) for Agreement Between RTA and Combined DA Scores for Each Case Reliability*: ICC < 0.40 is “poor”; ICC = 0.40 – 0.59 is “fair”; ICC = 0.60 – 0.74 is “good”; ICC > 0.74 is “excellent”; 95% CI LL = 95% confidence interval lower limit; 95% CI UL = 95% confidence interval upper limit. n: number

Case	n	ICC	95% CI LL	95% CI UL	Reliability*
1	41	0.67	0.46	0.81	Good
2	39	0.85	0.73	0.92	Excellent
3	39	0.50	0.23	0.70	Fair

**Table 5:** Milestone Assessment Variance Components ID: Variance attributable to residents; Rater: Variance attributable to raters; Residual: Variance attributable to other factors, plus random error.

Variance Component	Variance Estimate (Percentage Variance (%))		
	Case 1	Case 2	Case 3
ID	0.28 (44)	0.61 (75)	0.21 (47)
Rater	0.10 (15)	0.04 (4)	0.04 (8)
Residual	0.27 (41)	0.17 (21)	0.21 (45)

## Discussion

The design and results of this study confirm that DA of the ACGME milestone sub-competencies, using video recordings, produces scores that are comparable to RTA scores. In all three scenarios, the residents’ performance scores from the two different assessment methods were comparable both on average scores obtained and in terms of variance. These results affirm that milestone assessment in a simulation environment can be reliably performed using video capture and delayed scoring.

Video-based DA of the resident simulation performances for milestone evaluations offers several educational and logistical advantages over RTA. DA allows evaluations to be done at any convenient alternative time (e.g., during “administrative,” “nonclinical,” or “off-service” time) instead of during clinical work hours. This approach reduces the need to remove faculty from clinical duties during high-volume clinical hours, which subsequently decreases personnel costs to the department. Additionally, video recordings can be archived for future review to document progression over time, marking a resident’s milestone advancement. For instance, program directors and clinical competency committees can observe the performance of a resident in a simulated scenario addressing a particular patient care milestone during a resident’s early years in training and subsequently compare it to their performance at a later point in training.

To date, there are few studies that have investigated the use of video-based DA in medical trainee clinical evaluations. Moreover, some of the studies that employed video-based DA do not cite or include reliability data for their use of DA [6, 9]. Additionally, the studies comparing assessment results between RTA and DA focused only on the trainees’ technical skills, such as tissue-handling, pediatric rapid sequence intubation, joint examination, and laparoscopic surgical performance rather than interpersonal communication and professional competencies [22-26]. Our results are consistent with the existing literature that DA serves as a complement to RTA in the assessment of technical skills (Case 2). Furthermore, our results also suggest that DA is a promising method for evaluating more complex milestones competencies, such as crisis management (Case 1) and professionalism (Case 3).

The implications of the results in this study hold promise and value for any residency program struggling to balance patient care with clinical competency education and milestone evaluations. DA can decrease the logistical burden that a residency program faces if they use simulation sessions (e.g., OSCE) for milestone assessment. When multiple simulation scenarios for multiple residents occur simultaneously, as is the case in a large-scale session such as an OSCE, multiple clinical faculty are required to perform RTA. DA, however, reduces the need for clinical faculty with content expertise to be physically available at the time of the simulated patient care encounter, allowing them to perform other activities, such as patient care. Additionally, if employing a large scale OSCE, the DA method eliminates the need for multiple clinical faculty to perform simultaneous RTA.

There are limitations to this study. The patient care scenarios demonstrated a higher level of ICC than the professionalism scenario, despite our efforts to reach a consensus on specific behaviors that would be measured in a binary fashion (i.e., yes or no). The lower level of inter-rater agreement for the professionalism scenario may be due to the tight clustering of resident scores (Figure 1). As a result, the lack of variance in scores makes it more difficult for raters to show a consistent difference in scores between residents. Unfortunately, there is no single best method for evaluating professionalism in medical trainees [27-28]. Further, some studies show that inter-rater agreement for humanistic elements is often very low [29]. The evaluation of professionalism behaviors, unfortunately, requires some degree of subjectivity, so it is not surprising that the assessors in our study showed a lower level of ICC in comparison to the patient care scenarios. Additionally, the large proportion of variance attributable to other factors, plus random error, also indicates the need to include additional facets of assessment that varied among residents (e.g., the interaction between residents and raters), as well as obtaining more heterogeneous samples to address the issue of restricted sample variance [30]. This result also relates to another limitation of this study in that only residents from a single institution and in a single specialty domain participated in the study.  

Future directions

The ACGME milestones are based on six core competencies. In this study, we evaluated residents in two of these competencies: patient care and professionalism. In the future, we plan to assess scores obtained using DA for resident milestone performance in other competency domains, such as Interpersonal and Communication Skills and Practice-based Learning and Improvement. Additionally, future studies that include residents from multiple institutions and multiple medical specialties will help investigate the broader application of delayed assessment.

## Conclusions

In conclusion, this study demonstrates that the effective use of video recordings to assess ACGME milestones in a delayed manner holds promise as a reliable and logistically appealing method of scoring. Based on a department’s needs, our findings support the use of either real-time or delayed video evaluation for assessing milestones in a simulated patient care environment. Future studies should include scenarios and trainees from a variety of medical specialties. Further investigation is also needed to include milestones from all six core competencies areas.

## Appendices

Appendix 1: Example of an OSCE scenario and the corresponding observable behavior/action requirements to achieve the anesthesiology milestone competency. A) Case Description for Patient Care 5 – Crisis Management in the setting of a scenario entitled: “Trauma Resuscitation”; B) Milestone rubric map linked to the observable behavior/action in the context of the scenario “Trauma Resuscitation”.

1A) Case description: A 42-year-old male who sustained a head-on motor vehicle accident two hours ago presents directly from the emergency department (ED) to the operating room for an emergent exploratory laparotomy. He went to an outside hospital first and had bilateral chest tubes and two 16-gauge peripheral intravenous (PIV) catheters placed. He was given two units of packed red blood cells (PRBC) and was transferred here for further care. In the ED, he received an arterial line and a focused assessment with sonography for trauma (FAST) scan of his abdomen showing free fluid requiring emergent surgery. Your task is to induce anesthesia and manage the patient’s intraoperative course. 

1B)

**Table 2:** Example of the Conversion of an ACGME Sub-competency Milestone Rubric into an Analytical Checklist for Assessment in the Simulated Patient Encounters ACGME: Accreditation Council for Graduate Medical Education; IV: intravenous; ddx: differential diagnosis; ACLS: advanced cardiac life support

Milestone Level	ACGME Rubric	Observable Behavior/Action
Level 1	Recognizes acutely ill or medically deteriorating patients; initiates basic medical care for common acute events; calls for help appropriately	Identifies vital signs changes
Level 2	Constructs prioritized differential diagnoses that include the most likely etiologies for acute clinical deterioration; initiates treatment with indirect supervision and seeks direct supervision appropriately	Identifies at least two differentials for the vitals signs changes (blood loss, hypovolemia, transfusion reaction, shock, anaphylaxis, etc.) Gives IV fluids, blood, IV vasopressors (boluses or drips), or other treatments of ddx.
Level 3	Identifies and manages clinical crises with indirect supervision; may require direct supervision in complex situations	Recognizes that patients require ACLS Calls for help Performs some steps (Chest compressions, give code drugs, defibrillates) of ACLS without “help”
Level 4	Identifies and manages clinical crises appropriately with conditional independence; assumes increasing responsibility for leadership of crisis response team	Directs surgeon and nurse in ACLS as team leader Correctly performs all steps of ACLS
Level 5	Coordinates crisis team response	Coordinates the care of the perioperative team during the code.
